# Trkb Signaling in Pericytes Is Required for Cardiac Microvessel Stabilization

**DOI:** 10.1371/journal.pone.0087406

**Published:** 2014-01-31

**Authors:** Agustin Anastasia, Katrin Deinhardt, Shiyang Wang, Laura Martin, Donna Nichol, Krithi Irmady, Jasmine Trinh, Luis Parada, Shahin Rafii, Barbara L. Hempstead, Pouneh Kermani

**Affiliations:** 1 Department of Medicine of Weill Cornell Medical College, New York, New York, United States of America; 2 Department of Molecular Neurobiology, Skirball Institute, New York, New York, United States of America; 3 Centre for Biological Sciences and Institute for Life Sciences, University of Southampton, Southampton, United Kingdom; 4 Cell and Developmental Biology of Weill Cornell Medical College, New York, New York, United States of America; 5 Department of Developmental Biology, University of Texas Southwestern Medical Center, Dallas, Texas, United States of America; 6 Howard Hughes Medical Institute, Ansary Stem Cell Institute, and Department of Genetic Medicine, Weill Cornell Medical College, New York, New York, United States of America; University of Washington, United States of America

## Abstract

Pericyte and vascular smooth muscle cell (SMC) recruitment to the developing vasculature is an important step in blood vessel maturation. Brain-derived neurotrophic factor (BDNF), expressed by endothelial cells, activates the receptor tyrosine kinase TrkB to stabilize the cardiac microvasculature in the perinatal period. However, the effects of the BDNF/TrkB signaling on pericytes/SMCs and the mechanisms downstream of TrkB that promote vessel maturation are unknown. To confirm the involvement of TrkB in vessel maturation, we evaluated TrkB deficient (*trkb* ^−/−^) embryos and observed severe cardiac vascular abnormalities leading to lethality in late gestation to early prenatal life. Ultrastructural analysis demonstrates that *trkb^−/−^* embryos exhibit defects in endothelial cell integrity and perivascular edema. As TrkB is selectively expressed by pericytes and SMCs in the developing cardiac vasculature, we generated mice deficient in TrkB in these cells. Mice with TrkB deficiency in perivascular cells exhibit reduced pericyte/SMC coverage of the cardiac microvasculature, abnormal endothelial cell ultrastructure, and increased vascular permeability. To dissect biological actions and the signaling pathways downstream of TrkB in pericytes/SMCs, human umbilical SMCs were treated with BDNF. This induced membranous protrusions and cell migration, events dependent on myosin light chain phosphorylation. Moreover, inhibition of Rho GTPase and the Rho-associated protein kinase (ROCK) prevented membrane protrusion and myosin light chain phosphorylation in response to BDNF. These results suggest an important role for BDNF in regulating migration of TrkB-expressing pericytes/SMCs to promote cardiac blood vessel ensheathment and functional integrity during development.

## Introduction

During late embryonic development, the formation of mature and fully functional blood vessels depends on the tightly regulated association of endothelial cells and mural cells such as pericytes and smooth muscle cells (SMCs). Many growth factors, such as vascular endothelial growth factor (VEGF), regulate endothelial cell migration and survival whereas platelet derived growth factor (PDGF) is involved in the regulation of remodeling and maturation of blood vessels via actions on pericytes and smooth muscle cells [1].

Pericytes/SMCs are adventitial cells located within the basement membrane of capillaries and post-capillary venules. These contractile cells play an important role in stabilizing nascent endothelial tubes by providing essential survival factors [2], inhibiting endothelial cell proliferation, and guiding vessel wall remodeling in response to growth factors [3]. Pericytes/SMCs are intimately associated with endothelial cells through the extension of cytoplasmic processes. Reciprocal interactions between endothelial cells and pericytes/SMCs have been well characterized in terms of growth factor-receptor signaling by PDGF. PDGF is expressed by endothelial cells and binds to PDGF receptor β (PDGFRβ) on the surface of developing pericytes in immature blood vessels. Genetic deletions of *pdgf* or *pdgfrβ* result in perinatal lethality, as a consequence of vascular dysfunction caused by mural cell deficiency [4], [5]. However, the molecular mechanisms that regulate the recruitment of pericytes/SMCs, and the extension of pericyte processes to provide coverage of microvascular endothelial cells and vascular integrity are incompletely understood.

Numerous studies have described critical roles for neurotrophins and their receptors in non-neuronal cells, such as endothelial cells, smooth muscle cells, immune cells, and epithelial cells in different organs [6]–[9], [11]. BDNF deficiency results in reduction in endothelial cell-cell contacts and in endothelial cell apoptosis, whereas BDNF overexpression results in increased capillary density, establishing the essential role of BDNF in modulating cardiac microvascular endothelial cells during cardiac development [10]. More recent studies confirm that BDNF mediates these effects during development by activating its receptor tyrosine kinase TrkB [11]. BDNF plays a critical role in regulating both vascular development and the vascular response to injury. Unlike VEGF-A, which activates the receptors VEGFR1 and VEGFR2 expressed on most endothelial cell populations and is critical for early stages of vascular development, BDNF is expressed in an organ-specific manner, restricted to the heart and skeletal muscle vasculature during the perinatal period [10]. Endothelial cells lining arteries and capillaries of the heart express BDNF, first detectable in mid to late gestation and maintained into adulthood. TrkB expression has been localized to perivascular cells in the developing heart (E18.5), and in the smooth muscle cell layer of coronary vessels [10]. Mice deficient in BDNF (*bdnf ^−/−^*) exhibit impaired survival of endothelial cells in intramyocardial arteries and capillaries in the late gestational and early postnatal periods [10]. Vascular hemorrhage in neonatal *bdnf ^−/−^* mice is restricted to cardiac vessels, reflecting the localized expression of BDNF and its receptor TrkB in the cardiac and skeletal muscle vasculature [10]. Genetic disruption of *trkb* leads to lethality during late embryonic development [11], [12], but the cause of the early death is unknown.

Here we demonstrate that TrkB is required for the development of the cardiac microvasculature, and *trkb^−/−^* embryos exhibit a specific deficiency in pericytes/SMCs. Ultrastructural analysis shows that *trkb^−/−^* embryos have defects in endothelial cell integrity including discontinuity of the cell membrane, leading to perivascular edema. We also determined that pericyte/SMC-specific deletion of TrkB results in abnormalities of vasculogenesis that significantly contribute to the lethal phenotype observed in *trkb^−/−^* mice. Conditional TrkB deletion in SMCs results in marked reduction of pericyte/SMC density in the heart and perinatal lethality. Using cultured SMCs, we further identified that BDNF/TrkB signaling leads to cell migration and contractile force generation by actomyosin phosphorylation, to play a pivotal role in regulating pericyte morphology and migration.

## Materials and Methods

### *Trkb* Null and *trkb* Floxed Animals

Heterozygous *trkb (+/−)* mice (Jackson Laboratories, Jax Stock #003098) on a C57Bl/6 background were intercrossed to generate homozygous animals. For embryo analysis, the gestational age was determined by morphological criteria including limb bud, eye development, length, and weight of the embryo. The genotypes of embryos or neonates were determined using tail-derived DNA and PCR amplification as described by Jackson Laboratories. *Trkb* floxed (*trkb^f/f^*) animals were generated as described previously [13]. Briefly, exon 1 of the *trkb* gene, which encodes the signal peptide and the first 40 amino acids of the N terminus of TrkB, was floxed. Crossing a *trkb ^f/f^* mouse with a transgenic mouse carrying Cre driven by a smooth muscle actin promoter (SMC-Cre, Jax Stock #004746), generated progeny in which the expression of *trkb* was selectively eliminated in perivascular cells including pericytes/SMCs. The genotype of each animal was assessed using PCR of genomic DNA isolated from tail, utilizing trkB.flox forward primers 5′ TGG TGT ACT GAG CCT TCT CCA G 3′ and trkB.flox reverse primers 5′ CGA GGC TTG GAG TTG ACT GGT 3′. All animal procedures of this study were approved by the IACUC of Weill Cornell Medical College and all efforts were made to minimize animal suffering.

### Tissue Analysis

Following sacrifice, embryos were immediately fixed in 3% paraformaldehyde in PBS for 18 hours. Dissected tissues were embedded in paraffin for histological analysis for hematoxylin-eosin staining, or infiltrated with 30% sucrose in PBS prior to cryoprotection in 30% sucrose:OCT (1∶1; Tissue Tek, Sakura, CA) for immunohistochemical analysis. For electron microscopic analysis, embryos and newborn mice were sacrificed by decapitation and hearts were immediately removed from the chest cavity and fixed in Karnovsky’s fixative solution for 18 hours prior to embedding in Epon. One µm sections were stained with toluidine blue for initial evaluation, followed by ultra-thin sections that were counterstained with lead citrate and viewed with a JEOL electron microscope. To analyze vascular permeability, FITC-dextran (70 kD; Sigma) was injected as a bolus through the tail vein of 3-month-old mice. After perfusion for 10 minutes, the animals were sacrificed and hearts harvested and processed for fluorescence microscopy. FITC-dextran intensity was quantified by densitometry using ImageJ software.

### qRT-PCR

RNA from the hearts of 3 to 6 week old mice was extracted using RNeasy Plus Mini Kit (Qiagen) according to the manufacturer’s instructions. cDNA from total RNA was generated using random hexamers and Superscript III (Invitrogen). qPCR for the mouse *trkb* gene was performed using PerfeCTa SYBR Green FastMix ROX (Quanta Biosciences) and the thermocycler ABI Prism 7900 HT (Applied Biosystems). At least 5 mice of each genotype were evaluated using primers that annealed to sequences in exon 5 (5′-ACTGCCTCAATGAGAGCAGC-3′) and exon 6 (5′-GGAAAGGGTCACAGACTTTCC-3′), present in all *trkb* transcripts. The level of expression of *trkb* mRNA in gene-targeted animals was calculated as the fold change relative to the expression level of *trkb* in the corresponding wild type littermate. Relative mRNA expression level for each sample was normalized to the *actin* gene and 18S ribosomal RNA as reference genes.

### Immunohistochemical Analysis

For postnatal tissue, 10 micron serial frozen sections were stained with NG2 antibody (Cat# AB5320, Millipore, MA) or CD31/PECAM (BD Pharmingen, CA, clone MEC13.3, 5 µg/ml) to detect pericytes/SMCs or endothelial cells, respectively. We used isolectin B4 (Cat# B-1205, Vector Laboratories) to identify endothelial cells and PDGFRβ (Cat# AB91066, Abcam) for pericytes/SMCs in embryos. The TrkB antibody was purchased from Millipore (Cat# 07–225). The collagen IV antibody was from Abcam (Cat# Ab6586). Collagen intensity was quantified by densitometry using ImageJ software. For double-immunofluorescence, frozen sections were incubated with primary antibodies overnight, followed by incubation with either Alexa 488 or 568 conjugated secondary antibodies (Invitrogen, NY) at room temperature and visualized using a Zeiss microscope. Fluorescence field images were captured digitally (Obzerver.Z1 microscope, AxioCamMrm camera, Zeiss). Counts of NG2 or PDGFRβ and PECAM objects in the heart were performed after unbiased intensity and size selection criteria. A gray level threshold was selected for all the microphotographs of all different treatments and staining. After this selection, ImageJ (Image J 1.34j; US National Institutes of Health, Bethesda, MD, USA, (http://rsb.info.nih.gov/ij/) particle analysis plug-in was used to set the lower and higher size of pixels to be considered as a countable object. Both selection thresholds were arbitrarily defined, but maintained rigorously for all the images of different conditions. Finally, objects were counted in each image using the ImageJ cell counter plug-in. NG2, PDGFRβ and PECAM intensity was analyzed by densitometry. To further ensure absence of bias in object counting or densitometry analysis, sections were coded, and the operator was blind to treatment groups. Negative controls without primary antibodies were performed for each immunodetection.

### Scratch-migration Assay

Human umbilical smooth muscle cells (HuSMCs) were isolated as described previously [14] and cultured in DMEM with 10% fetal bovine serum (FBS). HuSMCs were obtained through Institutional Review Board-approved protocols (Weill Cornell Medical College). HuSMCs (passage 7) were seeded in duplicate on 6-well plates at 1×10^6^ cells per well, and grown to confluency. Cells were incubated at 37°C in 5% CO_2_. Prior to treatment, cells were placed in DMEM without FBS for 18 hours. A vertical scratch through the plate was made gently using a 1 ml pipette tip. Following scratching, plates were washed with media to remove floating cells and debris. BDNF (100 ng/ml) was added to fresh media without FBS and cells were photographed at 6, 13 and 24 h followed by area analysis of the scratch using ImageJ. The width and area of each scratch in each condition was analyzed in 7–10 random and separate fields (phase contrast, 5X, microscope Zeiss Axiovert 200 M).

### Live Imaging using Time-lapse Differential Interference Contrast (DIC) Microscopy

HuSMCs were plated on glass bottom dishes (MatTek Corporation) and cultured in DMEM containing 10% FBS for 24 hours. Medium was changed to serum-free DMEM overnight. Cells were then imaged in phenol red-free DMEM (Invitrogen) supplemented with 30 mM HEPES-NaOH (pH 7.4) using an Olympus IX71 inverted microscope driven by IPLab software (BD Biosciences) and equipped with a 60X PlanApoN objective (NA 1.42), a Hamamatsu EM-CCD camera and a heated stage maintained at 37°C. Images were captured every 15 seconds. Cells were treated with BDNF (100 ng/ml). For inhibitor studies, cells were pre-treated with 10 µM Y-27632 (Cat# 688001, Millipore) or 10 nM K-252a (Cat# 420297, Millipore) for 45 minutes, or pre-incubated with 1 µg/ml C3 transferase (Cat# CT04, Cytoskeleton Inc.) for 4 hours. All movies were exported and processed using IPLab (BD Biosciences) and ImageJ (NIH) software.

### Western Blot Analysis

HuSMCs were lysed in TNE buffer (10 mM Tris, pH 7.8, 1% Nonidet P-40, 0.15 M NaCl, 1 mM EDTA, protease inhibitor cocktail (Sigma) and phosphatase inhibitors (Sigma)) for 30 minutes at 4°C. 100 µg of total cell lysate protein was loaded on SDS-polyacrylamide gel and proteins were resolved and transferred to PVDF membrane. The following primary antibodies were used: TrkB (Cat# 07–225, Millipore), AKT (Cat# 46915, Cell Signaling Technology), phospho-AKT (Cat# 92715, Cell Signaling Technology), ERK (Cat# 46955, Cell Signaling Technology), phospho-ERK (Cat# 57265, Cell Signaling Technology), MLC (Cat# 3672, Cell Signaling Technology), and phospho-MLC (Cat# 3674, Cell Signaling Technology) antibodies. Anti β-actin (Cat# A-5441, Sigma) was used as a loading control. The respective secondary antibodies (anti-rabbit or anti-mouse IgG) were conjugated with HRP. Chemiluminescence of the blots was detected using ECL Advance Western Blotting Detection kit.

### TrkB Knockdown using shRNA Lentiviral Vectors

Lentiviral plasmids containing shRNA against human TrkB (targeting sequence: AATGCTCGCAAGGACTTTCAT; a kind gift of Dr. Simon S. Murray, The University of Melbourne, Australia) and a scramble control were obtained from Open Biosystems Thermo Scientific (Waltham, MA, USA). Lentivirus was packaged by co-transfection of shRNA constructs with packaging plasmids pMD2.G and pCMV-dR8.9 using TransIT-LT transfection reagent (Mirus, Madison, WI, USA) into HEK293FT cells. Media was changed 24 h later, and the media supernatant was collected 48 h after transfection. Supernatants were filtered through a 0.45-mm filter and pelleted by centrifugation with PEG-it (System Biosciences, Mountain View, CA, USA). Pellets were resuspended in medium. HuSMCs were infected, 72 h later media was changed to serum free, followed by BDNF treatment (100 ng/ml). The efficacy of TrkB knockdown was assessed by Western blot.

## Results

### *trkb* Gene Deletion Results in Embryonic Lethality and Cardiac Vascular Defects

**Figure 1:**
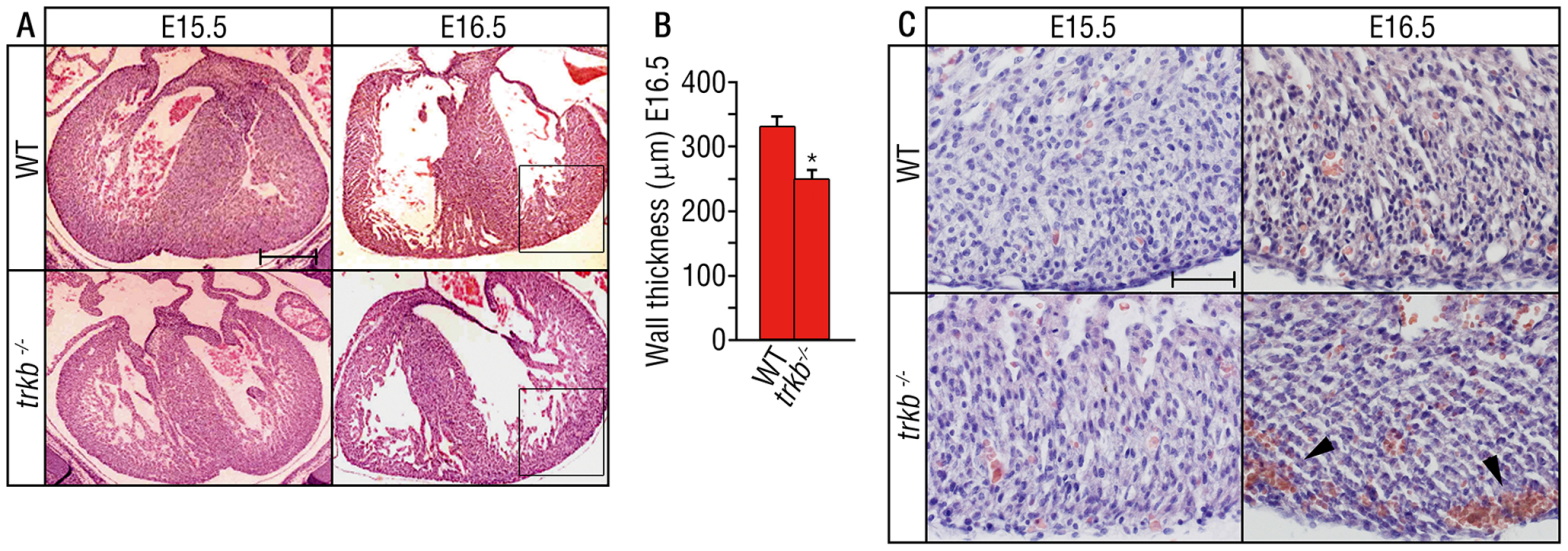
Ventricular wall thinning and hemorrhage in *trkb^−/−^* embryos. Embryos at the indicated gestational age were harvested from *trkb^+/−^* females. (A) Sections of the heart were analyzed following hematoxylin and eosin staining. Ventricular wall size was normal in mid-gestation in the wild type animals, but thinning became apparent by E16.5 in the *trkb^−/−^* embryos. The square inset represent the region were the ventricular wall was quantified in (B). Scale bar = 300 µm. (B) Quantification of (A). Bars represent mean ± s.e.m. n = 3 per group, where we analyzed 6 sections per animal. Statistical comparisons were made by one-way analysis of variance test. *P<0.05. (C) Sections of the right ventricular wall were analyzed histologically following hematoxylin and eosin staining. Intramyocardial hemorrhage from *trkb^−/−^* embryos could be detected at E16.5 (arrowheads) and was absent in wild type littermates. Scale bar = 50 µm.

BDNF and TrkB are expressed in the developing cardiac vascular bed with BDNF expressed by endothelial cells and TrkB expressed prominently by pericytes and vascular smooth muscle cells (SMCs) [10]. Furthermore, mice deficient in BDNF *(bdnf ^−/−^)* exhibit severe cardiac vascular defects, leading to early postnatal mortality [10]. To determine whether TrkB mediates these effects, we generated *trkb^−/−^* embryos and observed late gestational lethality as early as embryonic day 17.5 (E17.5), consistent with a prior report [12]. Defects within the heart and vascular system are frequently responsible for *in utero* lethality of the embryo and early fetus [15]. To identify the critical actions of TrkB in the development of the cardiac vasculature prior to lethality, we initially examined the cardiac morphology from *trkb^−/−^* or wild type littermates during late gestation. Histological analysis of the hearts from E15.5 to E16.5 embryos showed reduced ventricular wall thickness from the *trkb^−/−^* embryos as compared to wild type littermates, most prominently in the left ventricular wall (Figure 1A, B). There were no significant differences in overall heart size or the anatomical structure of the heart (Figure 1A). Moreover, the great vessels appeared normal in *trkb^−/−^* compared to wild type littermates. This phenotype is similar to that reported for the *bdnf ^−/−^* mouse [10]. However, the onset of intravascular hemorrhage was detectable prenatally in *trkb* deficient embryos, whereas it occurs postnatally (P0–P4) in mice with *bdnf* deficiency [10].

The ability of *trkb^−/−^* embryos to survive until late gestation and to form identifiable cardiac arteries, capillaries, and veins suggest that the earlier processes of vasculogenesis and sprouting angiogenesis are not greatly impaired. To establish the onset of intramyocardial vessel defects, *trkb^−/−^* embryos and wild type littermates were examined from E14.5 to P0. Intramyocardial hemorrhage in *trkb^−/−^* embryos could be detected first at E16.5 (Figure 1C) and was present in E17.5 embryos (data not shown), but it was absent in wild type littermates. The onset of hemorrhage in late gestation suggests that TrkB is not required for the initial stages of cardiac vasculogenesis and sprouting angiogenesis as gene-targeted embryos with these defects typically die *in utero* between embryonic days 9 and 13 [15].

**Figure 2:**
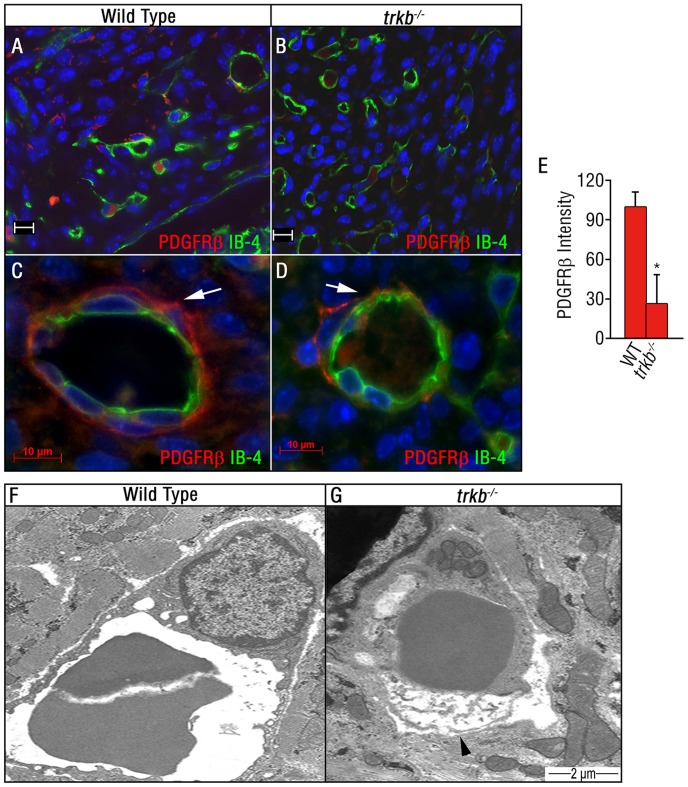
Microvessel abnormalities in *trkb^−/−^* embryos. (A–D) Immunofluorescent staining using IB-4 (green) to detect endothelial cells and PDGFRβ (red) to detect pericytes, within the ventricular wall in wild type (A–C) and *trkb^−/−^* (B–C) littermates at E17.5. High magnification images at 100X for wild type (C) and *trkb^−/−^* (D). The PDGFRβ staining is discontinuous and lacks close apposition to IB-4 in the *trkb^−/−^* embryos (arrow) as compared to the wild type embryos. Scale bar A, B = 20 µm. Scale bar C, D = 10 µm. (E) Quantification of (A–D). Bars represent mean ± s.e.m. n = 3 per group. Statistical comparisons were made by one-way analysis of variance test. *P<0.05. PDGFRβ immunoreactivity in the *trkb^−/−^* hearts is reduced, as compared to the wild type hearts. (F,G) Electron-micrographs of ventricles from wild type (F) or *trkb^−/−^* (G) embryos at E17.5. Endothelial cells frequently exhibited degenerated plasma membrane (arrow head) and perivascular edema was consistently detected in capillaries and arterioles of *trkb^−/−^* embryos. Scale bar F,G = 2 µm.

We next investigated whether TrkB is required for the development of the cardiac microvasculature. We compared the vascular cell density in *trkb* ^−/−^ and wild type embryos assessed by double-immunolabeling for endothelial cells and pericytes/SMCs using isolectin B4 (IB4) and PDGFRβ antibodies, respectively. At E17.5, PDGFRβ-positivity was significantly reduced in the left ventricular wall of *trkb^−/−^* embryos as compared to wild type littermate controls (Figure 2A, D), showing decreased perivascular coverage of the blood vessels *in vivo* (Figure 2E). To further characterize the defects in vessel morphology in *trkb^−/−^* mice, ultrastructural analysis was performed on wild type and *trkb^−/−^* E17.5 embryos. The majority of endothelial cells in arterioles and capillaries in heart sections from *trkb^−/−^* animals exhibited degeneration and discontinuity of the plasma membrane, detachment from the underlying basal membrane, and perivascular edema (Figure 2G). No abnormalities were noted in endothelial cells from wild type littermates (Figure 2F).

Together these results suggest that the establishment of the cardiac vascular network, and its patterning to form veins, arterioles, and capillaries, are not substantially perturbed in *trkb^−/−^* embryos. However, vascular hemorrhage in the heart is detectable from E16.5 onwards during the developmental time frame in which TrkB vascular expression normally commences [10]. In addition to vascular defects, *trkb^−/−^* embryos exhibited abnormalities in ventricular wall thinning by E16.5 (Figure 1A) that may be a secondary effect due to impaired blood flow.

### Pericyte-specific Deletion of TrkB Exhibits Reduced Pericyte Density

**Figure 3:**
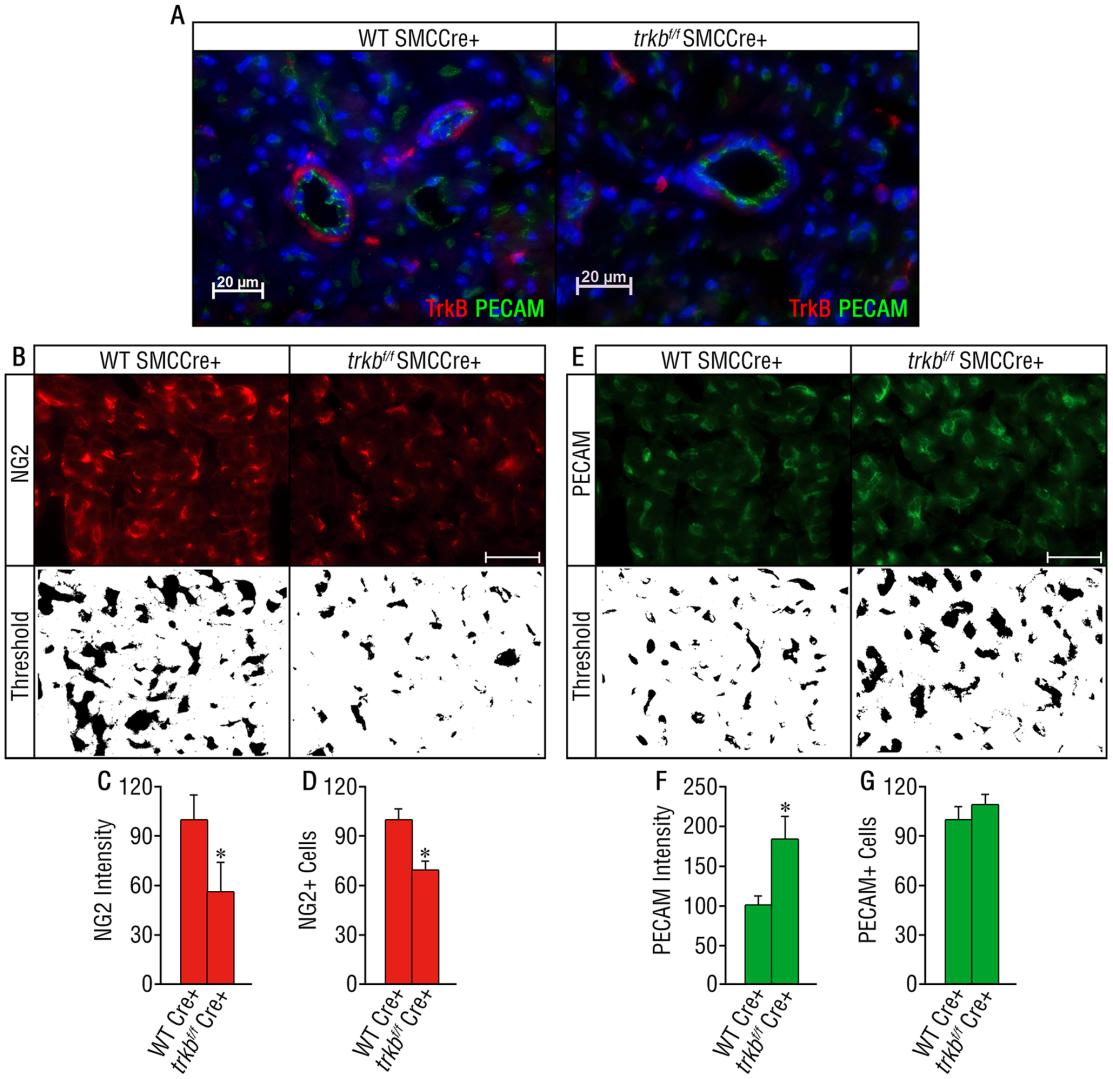
Pericyte/SMCs-specific deletion of TrkB induces a reduction in pericyte density. (A) Double immunofluorescence staining of the left ventricular wall from P21 mice. TrkB (red) is expressed by perivascular cells (PECAM in green) in wild type mice and is largely reduced in *trkb^f/f^-*SMCCre+ littermates. (B–G) Quantification of NG2+ (B, C, D) and PECAM+ (E, F, G) cells and intensity in the heart. *n = 3 per genotype.* Bars represent mean ± s.e.m. Statistical comparisons were made by one-way analysis of variance test. (C) *: P = 0.0023. (D) *: P = 0.007. (F) *: P = 0.0022. Scale bar A,B and E = 20 µm.

To investigate how TrkB modulates pericyte/SMC function during the development of the cardiac vasculature, we generated mice selectively deficient in *trkb* in pericyte/SMCs by crossing floxed *trkb* mice (*trkb^f/f^*) [13] with smooth muscle cell alpha actin (SM22a) Cre expressing mice (SMCCre). Heterozygous mutants were generated and further intercrossed to obtain homozygous embryos. *Trkb^f/f^*-SMCCre+ allele demonstrated 20% reduction in Mendelian inheritance (expected ratio: 25%; observed ratio: 20%, Table S1). In those *trkb^f/f^*-SMCCre+ embryos that survived, qRT-PCR analysis of the heart RNA showed ∼80% reduction of the TrkB transcript compared to wild type*-*SMCCre+ littermates at postnatal day 21 (Figure S1). In wild type mice, TrkB is expressed by perivascular cells surrounding platelet endothelial cell adhesion molecule positive (PECAM+) endothelial cells (Figure 3A). In contrast, TrkB expression was markedly reduced in the majority of pericytes/SMCs of the *trkb^f/f^*-SMCCre+, however, in a small subset of perivascular cells TrkB expression was unaffected by the conditional deletion (Figure 3A). This result suggests that pericytes/SMCs represent the major source of TrkB in the neonatal heart, and that TrkB expressed by pericytes promotes the survival of the embryos.

We next quantified pericyte coverage of the cardiac microvasculature utilizing dual NG2 proteoglycan (pericyte/SMC marker) and PECAM (endothelial marker) immunodetection to determine whether deletion of TrkB results in loss of pericytes/SMCs. At postnatal day 21, *trkb^f/f^-*SMCCre+ mice displayed a 50% reduction in NG2+ perivascular cell density compared to the wild type control mice (Figure 3B, C, D). Thus, the reduction in TrkB expression in pericytes/SMCs resulted in a reduction of the number of this cell population in developing vessels. However, the total number of capillaries is unchanged in the *trkb^f/f^-*SMCCre+ as shown by PECAM staining (Figure 3E, G), despite an increase in PECAM intensity in those cells (Figure 3F).

**Figure 4:**
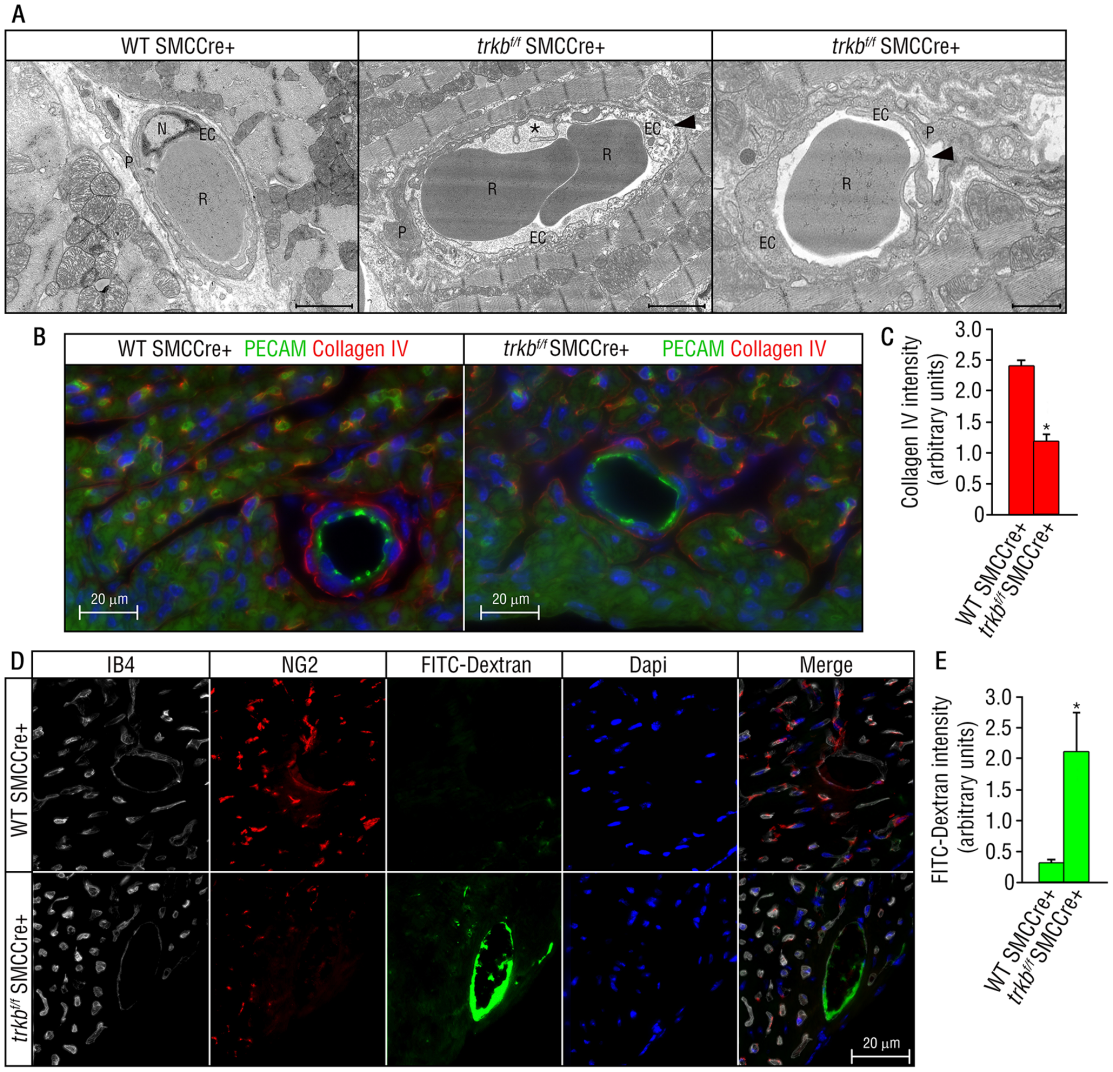
Vascular abnormalities in the *trkb^f/f^-*SMCCre+ mice. (A) Electron micrographs of hearts of wild type*-*SMCCre+ and *trkb^f/f−^* SMCCre+ mouse hearts at postnatal day 21. In the middle and right panel, cardiac capillaries show abluminal cytoplasmic protrusions (*), endothelial degeneration with evidence of loss of cell-cell contact, perivascular edema and detachment in arteries and capillaries (arrow head) in the *trkb^f/f^-*SMCCre+ mouse hearts as compared to control wild type-SMCCre+ littermates (left panel). Scale bar left and middle panel = 2 µm. Scale bar right panel = 1 µm. N: Nucleus; EC: Endothelial Cell; P: Pericyte; R: red cell. (B) Reduction in Collagen IV deposition surrounding arterioles and microvessels in the *trkb^f/f^-*SMCCre+ as compared to wild type-SMCCre+ hearts (PECAM in green and collagen IV in red). Scale bar = 20 µm. (C) Quantification of (B). Bars represent mean ± s.e.m. n = 3 per group. Statistical comparisons were made by one-way analysis of variance test. *P<0.05. (D) FITC-dextran and immunofluorescence analysis of sections corresponding to the left ventricular vasculature in *trkb^f/f−^* SMCCre+ or wild type-SMCCre+ littermates at 3 months of age. IB4+ endothelial cells in the far red channel (white), NG2+ pericytes/SMCs in the red channel, FITC-dextran in the green channel, and DAPI in the blue channel. Increased extracellular accumulation of the FITC-dextran dye around pericyte-deficient vasculature in the hearts of *trkb^f/f−^* SMCCre+ mice, compared to wild type-SMCCre+ littermate controls. *n = 3 per genotype.* Scale bar = 20 µm. *(E) Quantification of FITC-dextran in (D).* Bars represent mean ± s.e.m. n = 3 per group. Statistical comparisons were made by one-way analysis of variance test. *P<0.05.

To better understand the cellular basis of the vascular defects observed in surviving *trkb^f/f^-*SMCCre+ mice, we examined the cardiac vasculature at the ultrastructural level at postnatal day 21. In wild type mice, the endothelial abluminal surface was free of cytoplasmic extensions, and the basement membrane between endothelial cells and pericytes was intact (Figure 4A). In contrast, endothelial cells from *trkb^f/f^-*SMCCre+ mice exhibited loss of cell-cell contact, perivascular edema, and loss of apposition of endothelial cells and pericytes (Figure 4A). These ultrastructural defects are similar to those observed in the *trkb^−/−^* mice hearts, although not as profound. We further studied key vascular basement membrane components, and observed no significant changes in the deposition of endothelium-derived basement membrane proteins laminin γ1 and fibronectin in *trkb^f/f^-*SMCCre+ pericyte-deficient vessels (data not shown), but we detected a significant reduction in collagen IV deposition as compared with wild type-SMCCre+ mice (Figure 4B, C). These results suggest an impairment of basement membrane synthesis in the cardiac vasculature of *trkb^f/f^-*SMCcre+ mice. Lastly, perfusion studies using FITC-dextran (70 kDa.) in 3-month-old mice, demonstrated an increase in extracellular accumulation of the dye around the pericyte/SMC-deficient vasculature of the *trkb^f/f^-*SMCCre+ hearts (Figure 4D, E). Wild type*-*SMCCre+ mice were used as controls in which no dextran particles were found around arteries and capillaries of the hearts (Figure 4D, E). Taken together, these results strongly suggest that TrkB maintains cardiac microvascular homeostasis by recruiting pericytes/SMCs. The lack of pericytes/SMCs in the *trkb^f/f^-*SMCCre+ mice leads to vascular abnormalities noted by an increase in the accumulation of dextran in the perivascular area, which is in agreement with specific cellular defects observed by ultrastructural analysis and reduced collagen IV deposition.

### BDNF Regulates the Morphology and Migration of Pericytes/SMCs through TrkB Activation

**Figure 5:**
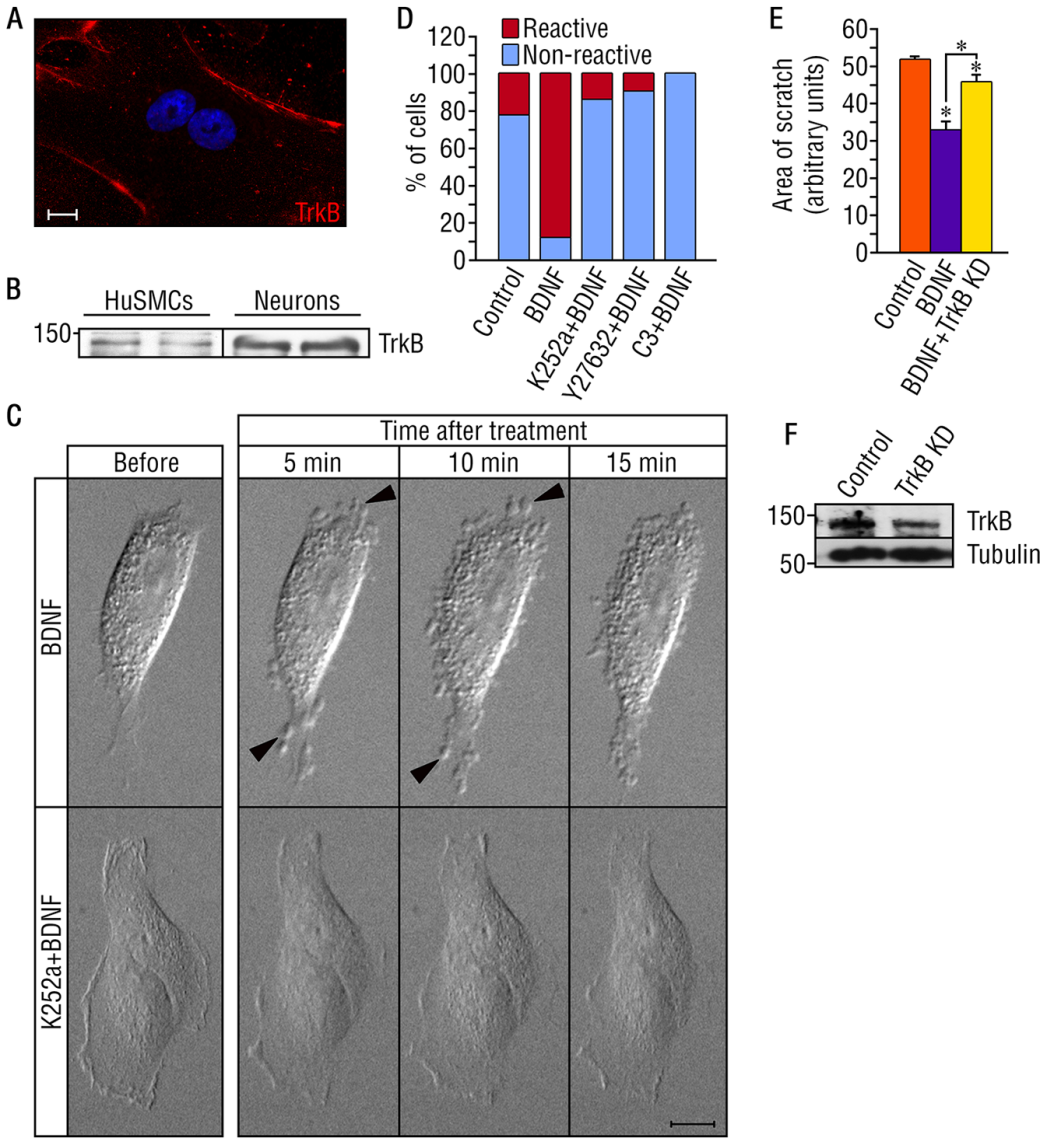
BDNF regulates the morphology and migration of human umbilical smooth muscle cells (HuSMCs). (A) Immunostaining of HuSMCs with TrkB antibody. Scale bar = 10 µm. (B) Western blot analysis for TrkB expression in HuSMCs. Lysate of rat cortical neurons in culture for 6 days was used as positive control for TrkB expression. (C) Phase contrast micrographs at different time points of HuSMCs treated with BDNF, and K252a+BDNF. and BDNF, The arrow heads represent membrane protrusions induced by BDNF. Scale bar = 20 µm. (D) Quantification of (C), and reactivity of HuSMCs after treatment with Y27632 (ROCK inhibitor) and BDNF, or C3 transferase (Rho inhibitor) and BDNF. n control = 9; n BDNF = 8; n K252a+BDNF = 7; n Y27632+BDNF = 10; n C3+BDNF = 7. (E) Scratch assay showing BDNF-induced HuSMCs migration 13 h after the addition of the ligand. The experiment was repeated in triplicate in 3 independent experiments. Bars represent mean ± s.e.m. Statistical comparisons were made by one-way analysis of variance test with Duncan’s *post hoc* analysis. *P<0.05. (F) Western blot analysis showing reduced expression of TrkB after shRNA knockdown.

The reduction in immunodetection of pericytes/SMCs in *trkb^−/−^* and *trkb^f/f^-*SMCCre+ mice coupled with the ultrastructural analysis suggesting a reduction in pericyte/SMC coverage allowed us to hypothesize that TrkB regulates pericyte/SMCs migration and/or ensheathment of endothelial cells. We investigated the role of TrkB in pericyte/SMC migration using cultured human umbilical smooth muscle cells (HuSMCs). HuSMCs express full length TrkB, as shown by immunohistochemical staining (Figure 5A) and western blot analysis (∼140 kD, Figure 5B). Truncated TrkB, which lacks tyrosine kinase activity, was not detectable in these cells (data not shown). Using *in vitro* live imaging, we observed acute morphological changes in HuSMCs in response to BDNF compared to untreated control cells. Within 2 to 5 minutes of BDNF addition, cells extend rounded cytoplasmic protrusions (Figure 5C; Movie S1). In tumor cells such as melanoma cells, cytoplasmic protrusions are associated with amoeboid fast migration, and are initiated by actomyosin contractility following RhoA activation [16]. BDNF-induced cytoplasmic protrusions were inhibited by pre-treatment with the Trk kinase inhibitor K252a (Figure 5C, D, Movie S2).

To directly evaluate the migration of HuSMCs in response to BDNF, we performed a scratch assay of cultured cells and assessed their migratory response to BDNF at multiple time points. BDNF induced the migration of HuSMCs as compared to control untreated cells at 6, 13 and 24 h (control scratch area in arbitrary units 6 h = 49.9±4.7; BDNF 6 h = 43.3±1.5; control 13 h = 48.8±6.0; BDNF 13 h = 36.4±1.6; control 24 h = 52.9±11.4; BDNF 24 h = 32.8±1.5; Figure 5E is showing 13 h of BDNF-induced migration). To confirm the involvement of TrkB in BDNF-induced migration, we infected HuSMCs with lentivirus expressing small hairpin RNA (shRNA) targeting TrkB. TrkB shRNA expression resulted in partial but significant decrease in TrkB protein levels as compared with the scrambled shRNA control infection (Figure 5E). HuSMCs expressing shRNA for TrkB exhibited reduced BDNF-induced migration (Figure 5E), a result that is in agreement with K252a inhibition of the BDNF-induced migration shown by live imaging *in vitro* (Figure 5C, D).

**Figure 6:**
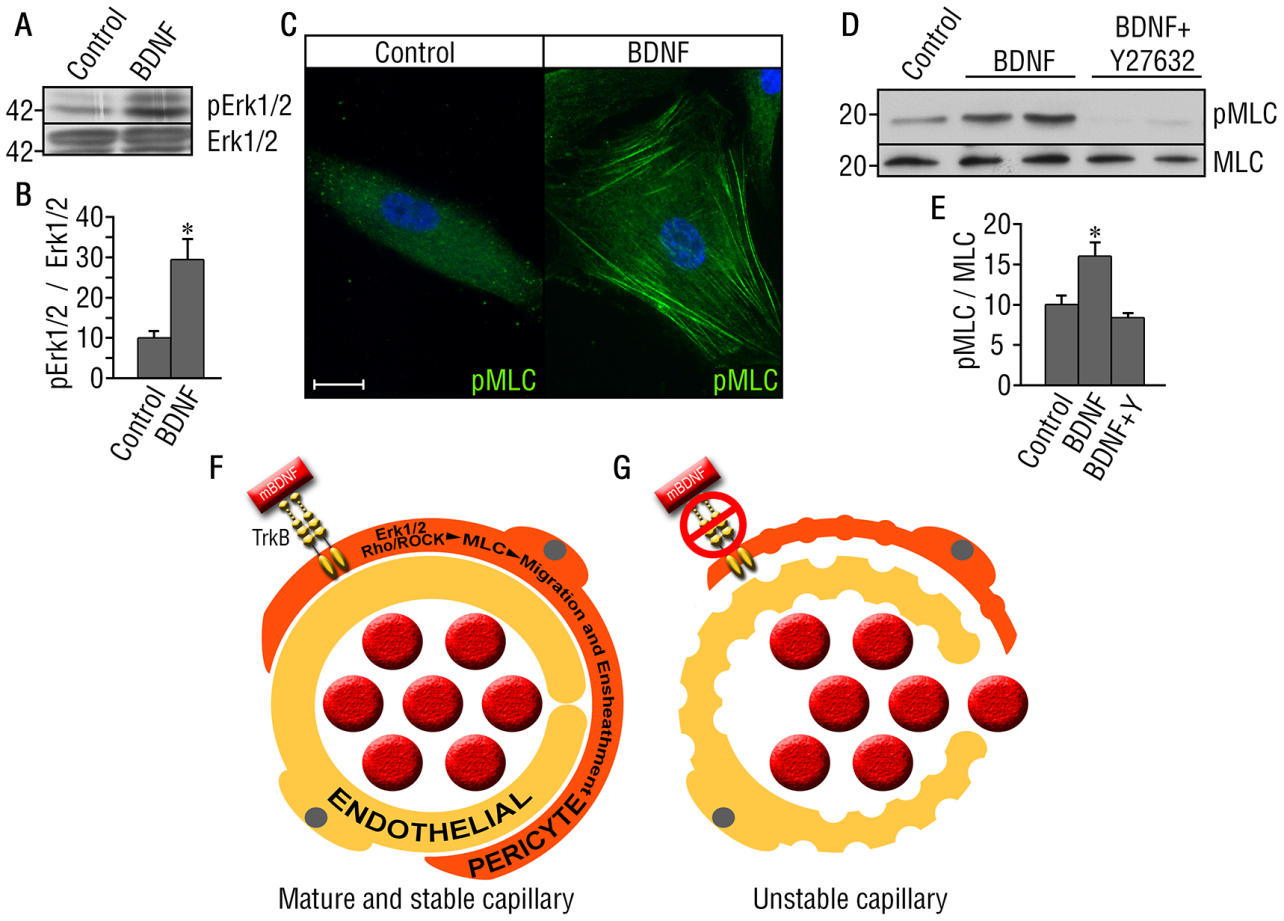
Signaling pathways involved in TrkB-BDNF-induced migration of HuSMCs. (A) ERK1/2 phosphorylation 30 minutes after BDNF administration to HuSMCs. (B) Quantification of (A) normalized to total ERK. Bars represent mean ± s.e.m. n = 3 per group. Statistical comparisons were made by one-way analysis of variance test. *P<0.05. (C) phosphor-MLC (green) after 1 hour of BDNF addition to HuSMCs. Representative images of 3 independent experiments. Scale bar = 20 µm. (D) Western blot showing MLC phosphorylation after 1 hour of BDNF addition (with or without the Y27632 ROCK inhibitor) to HuSMCs. (E) Quantification of (D) normalized to total MLC. Bars represent mean ± s.e.m. n = 3 per group. Statistical comparisons were made by one-way analysis of variance test with Duncan’s *post hoc* analysis. *P<0.05. (F–G) Proposed model of TrkB/BDNF signaling role in the cardiac microvasculature. (F) When TrkB/BDNF signaling is present, pericytes migrate in an ERK1/2, Rho/ROCK, and MLC-dependent manner to ensheath the endothelial cells in capillaries. This pericyte coverage of the endothelium promotes mature and stable capillaries. (G) In the absence of TrkB/BDNF signaling, pericytes do not cover the nascent vessels and the resulting capillaries are unstable and leaky.

To characterize the mechanisms by which BDNF/TrkB activation in pericytes/SMCs modulates their morphological changes and possible fast migration, we first evaluated the potential mediators of cell migration downstream of TrkB by examining the phosphoprotein profile in SMCs using the 4G10 antibody (pan-anti-phosphotyrosine). We observed phosphorylated proteins migrating at ∼42 kDda in the BDNF-treated HuSMCs as compared to untreated cells (data not shown). Reprobing of the membrane with specific antibodies, revealed that BDNF treatment induces activation of Ras/mitogen-activated protein kinases (ERK1/2) (Figure 6A, B). Klemke *et al*. (1997) have reported that the interaction of cells with growth factors in the extracellular matrix initiates ERK1/2 signaling leading to the activation of the motility machinery by enhancing phosphorylation of myosin light chain (MLC) [17]. Moreover, actomyosin contractility in many cell types results from phosphorylation of myosin light chain (MLC) downstream of the Rho kinases ROCK I and II [16]. Therefore, we performed pharmacological inhibition studies and observed that BDNF-induced cytoplasmic protrusions were inhibited by pre-treatment with the Rho-associated protein kinase (ROCK) inhibitor Y-27632, and the Ras homolog (Rho) inhibitor C3 transferase (Figure 5D), suggesting that this effect is a consequence of BDNF-TrkB signaling activation via the Rho/ROCK pathway.

To determine whether BDNF regulates actomyosin contractility in HuSMCs, we investigated the phosphorylation of MLC. Immunofluorescence analyses of phospho-MLC from unstimulated cells showed uniform and diffuse immunoreactivity, whereas HuSMCs treated with BDNF exhibited significant increase in phospho-MLC detection (Figure 6C). Analysis of phospho-MLC by Western blot demonstrated a marked increase in response to BDNF (Figure 6D, E). Furthermore, simultaneous treatment with BDNF and the ROCK inhibitor Y-27632 reduced MLC phosphorylation in the HuSMCs (Figure 6D, E). These results suggest that activation of TrkB by BDNF in HuSMCs activates the Rho/ROCK pathway to induce morphological changes associated with migration in this cell population.

Together, these findings suggest that BDNF-TrkB can promote pericyte/SMC migration via activation of actomyosin contractility involving ERK1/2 and Rho/ROCK pathways (Figure 6F, G). The significant decrease in pericyte coverage of endothelial cells observed in *trkb^f/f^-*SMCCre+ mice may therefore be a consequence of impaired migration of pericytes/SMCs, leading to inadequate vascular ensheathment.

## Discussion

Coronary blood vessel development requires BDNF, as mice deficient in this growth factor exhibit apoptosis of endothelial cells in the heart, leading to vessel destabilization and myocardial hemorrhage. These data raised the hypothesis that BDNF may act as an autocrine factor on endothelial cells or in a paracrine manner to induce activation of TrkB on neighboring pericytes, to stabilize nascent blood vessels. Here we establish the dependence of the cardiac vasculature on TrkB-mediated pericyte/SMC coverage of microvessels during development *in vivo* and SMCs migration *in vitro*. The lack of pericyte investment around endothelial cells in the cardiac capillaries and arterioles of TrkB deficient mice resulted in vascular leakage and ultimately in embryonic lethality. These studies identify a novel mechanism by which a growth factor:receptor system regulates in a paracrine manner the pericyte coverage, stability and homeostasis of the cardiac microvasculature.

During fetal embryogenesis, pericyte/SMC expressing PDGFRβ are recruited along the developing vascular endothelial tubes expressing PDGFB, results that are supported by *pdgfb* and *pdgfrβ* knockout mice which lack desmin-positive perivascular cells [4], [5]. Moreover, PDGFB expressed by the endothelium induces proliferation and migration of pericyte/SMC progenitors in early developmental stages. Later in development, blood vessels undergo vessel maturation, but the signals that govern this maturation are not well defined. Our data demonstrates that a subpopulation of PDGFRβ+ cells that co-express TrkB receptor are important to induce pericyte/SMC coverage which is essential for the blood vessel maturation and homeostasis in later stages of development. TrkB deficiency can explain the impaired recruitment of pericytes/SMCs to the nascent vasculature; however, other differences such as pericyte cell death in the *trkb^−/−^* mice may also have a role. Indeed, a previous report showed increased Tunnel labeling in the subepicardium of TrkB deficient embryos as compared to their wild type littermates at E12.5, although the identity of the apoptotic cells was not determined [11]. This report also showed a reduction of subepicardial capillaries in TrkB-deficient mice using markers for vascular endothelial cells [11]. Our studies extend this prior report by demonstrating a reduction of the left ventricular wall thickness in *trkb^−/−^* (Figure 1A, B) and endothelial cell degeneration by electron microscopy (Figure 2G).

Whereas developmental deletion of TrkB in all cells resulted in embryonic lethality as noted here and in prior studies [11], [12], conditional deletion of TrkB in pericytes/SMCs resulted in a ∼20% lethality of *trkb^f/f^*-SMCCre+ progeny. Notably, despite in the loss of coverage by cardiac pericytes, we found that 80% of the *trkb^f/f^-*SMCCre+ mice survived to adulthood and reached reproductive age. This could be explained by the partial decrease (∼80%) in *trkb* mRNA in the *trkb^f/f^*-SMCre+ mice heart (Figure S1). This incomplete deletion may be due to variable Cre recombination, but it has also been suggested that pericyte/SMC within the same organ arise from different embryonic origins [18], and we only have targeted a subset of pericytes/SMCs that express the transgelin promoter (smooth muscle protein 22-alpha). This could result in other pericytes/SMC compensating for the loss of the smooth muscle protein 22-alpha+ cells and promoting a less drastic phenotype compared with the *trkb* mice. Lastly, the TrkB receptor could be expressed on other non-vascular cells that can contribute to the unpredicted *trkb^f/f^*-SMCCre+ mice survival.

Our studies suggest that the extracellular matrix expressed by pericytes affects vascular stabilization. Extracellular matrix deposition regulates the transition from vascular morphogenesis to maturation [1]. Discontinuous or absent basement membrane can lead to blood vessel abnormality and instability. It has been shown that pericytes *in vitro* express numerous basement membrane components, such as collagen IV, laminin, and fibronectin. Studies by Stratman and colleagues [19] have shown that pericyte recruitment to endothelial cell-lined tubes during vasculogenesis is a stimulatory event controlling vascular basement membrane matrix assembly. Their study demonstrates an induction of the basement membrane components fibronectin, nidogen-1, perlecan, collagen IV, collagen VIII, and several laminin isoforms [19]. In accordance with their studies, our results show *in vivo* that a reduction in pericyte number leads to decreased deposition of collagen IV in mice with TrkB deficiency in pericytes/SMCs. These data suggest that active communication between pericytes and endothelial cells through BDNF/TrkB signaling pathways regulates extracellular matrix deposition.

Our results suggest that conditional TrkB deletion in pericytes/SMC impedes the recruitment of these cells to the microvasculature. To identify signaling pathways mediating these effects we used HuSMCs in culture and showed that BDNF-TrkB activation leads Rho GTPases activation (Rho/ROCK pathway), Erk1/2 and MLC phosphorylation and the consequently migration of the cells. Rho GTPases and MLC have been described as key regulators of pericytes/SMC shape and contractility [17], [20]–[21]. Furthermore, Kutcher and collaborators [22] have shown that the Rho GTPase-signaling pathway modulates capillary endothelial cell proliferative status and pericyte-endothelial cell interaction *in vitro* which is in agreement with our results.

Dysfunction in pericyte/SMC migration has been reported in many microvascular diseases, such as hypertension and diabetic microangiopathy. Therefore, it is of great importance to better understand the mechanisms by which pericytes regulate microvessel function.

## Supporting Information

Figure S1 **TrkB mRNA analysis in** ***trkb^f/f^*** **-SMCCre+ animals.** TrkB mRNA is markedly decreased in homozygous mice lacking *trkb* gene specifically in smooth muscle cells (*trkb^f/f^*-SMCCre+) compared to wild type-SMCCre+ control littermates. Bars represent mean ± s.e.m. n = 5 animals for each genotype, including mice from 3 different litters between the ages of 3 to 6 weeks old. Statistical comparisons were made by one-way analysis of variance test. *P<0.05.
(DOCX)

Table S1 ***Trkb^f/f^*** **-SMCCre+ genotype analysis.** Genotype analysis of 137 offsprings from *trkb^f/w^*-SMCCre+ heterozygous intercrosses at postnatal day 21. Numbers of animals of each genotype are shown with the correspondent percentage of the total mice alive at P21 in brackets.
(DOCX)

Movie S1 **Effect of BDNF on HuSMCs analyzed by live imaging.** Increased membrane protrusions in HuSMCs in the presence of BDNF.
(AVI)

Movie S2 **The effect of BDNF on HuSMCs was prevented by K252a.** BDNF-induced membrane protrusion in HuSMCs was inhibited by K252a.
(AVI)
