# Supplementary figures and images for: Trkb Signaling in Pericytes Is Required for Cardiac Microvessel Stabilization
Source: PLoS One. 2014 Jan 31;9(1):e87406. doi: 10.1371/journal.pone.0087406 (PMC3909185; doi:10.1371/journal.pone.0087406)

**Figure S1:**


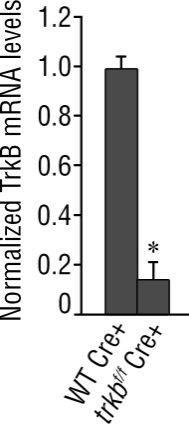

Supplement: Figure S1 — TrkB mRNA analysis in trkbf/f -SMCCre+ animals. TrkB mRNA is markedly decreased in homozygous mice lacking trkb gene specifically in smooth muscle cells (trkbf/f-SMCCre+) compared to wild type-SMCCre+ control littermates. Bars represent mean ± s.e.m. n = 5 animals for each genotype, including mice from 3 different litters between the ages of 3 to 6 weeks old. Statistical comparisons were made by one-way analysis of variance test. *P<0.05. (DOCX) [file pone.0087406.s001.docx]
