# Supplementary material for: Trkb Signaling in Pericytes Is Required for Cardiac Microvessel Stabilization
Source: PLoS One. 2014 Jan 31;9(1):e87406. doi: 10.1371/journal.pone.0087406 (PMC3909185; doi:10.1371/journal.pone.0087406)
Supplement: Table S1 — Trkbf/f -SMCCre+ genotype analysis. Genotype analysis of 137 offsprings from trkbf/w-SMCCre+ heterozygous intercrosses at postnatal day 21. Numbers of animals of each genotype are shown with the correspondent percentage of the total mice alive at P21 in brackets. (DOCX) [file pone.0087406.s002.docx]

**Table S1:**

|  | Genotypes | | |
| --- | --- | --- | --- |
|  | *trkb^f/w^*-Cre+ | *trkb^w/w^*-Cre+ | *trkb^f/f^*-Cre+ |
| Observed | 76 (55%) | 34 (25%) | 27 (20%) |
| Expected | 50% | 25% | 25% |
